# Treating comorbidities in MS is disease modifying: Commentary

**DOI:** 10.1177/13524585251338755

**Published:** 2025-05-03

**Authors:** Raffaele Palladino

**Affiliations:** Department of Public Health, University ‘Federico II’ of Naples, Naples, Italy; Department of Primary Care and Public Health, Imperial College of London, London, UK; Interdepartmental Research Center on Management and Innovation in Healthcare, University ‘Federico II’ of Naples, Naples, Italy

People with multiple sclerosis (PwMS) exhibit a higher prevalence of comorbidities, particularly cardiovascular, psychiatric, and autoimmune disorders, as compared with the general population.^1–3^ These comorbid conditions are frequently undertreated and have been linked to increased relapse rates, accelerated disability progression, diminished health-related quality of life, and heightened mortality.^1,2,4,5^ Given this substantial impact, an important question arises: could effective comorbidity management, including risk factor control, exert disease-modifying effects in MS? This pivotal issue is explored in depth in the Controversy section of this edition of the *Multiple Sclerosis Journal*.

Lorefice and Zivadinov argue that the current MS treatment paradigm, which primarily focuses on immunosuppression, fails to address the significant role of comorbidities in disease progression. Cardiovascular, metabolic, psychiatric, and autoimmune conditions exacerbate neurodegeneration through systemic inflammation, oxidative stress, and endothelial dysfunction.^6,7^ Despite evidence linking hypertension, dyslipidemia, and diabetes to accelerated MS progression,^7^ these conditions remain separate from disease-modifying strategies. When selecting the appropriate disease-modifying treatments (DMTs), MS neurologists should consider that some exhibit cardiovascular benefits,^7^ but others may negatively impact metabolic health,^8^ highlighting the need for integrated management. Furthermore, psychiatric and autoimmune comorbidities also influence disease outcomes,^7^ yet their treatment remains largely adjunctive rather than central to MS care. Beyond pharmacological interventions, optimizing vascular health, cognitive reserve, and brain resilience is crucial for mitigating disability accumulation. The commentary calls for a fundamental shift in MS treatment, advocating for comorbidity-targeted interventions as a core component of disease modification, rather than mere supportive care.

In the other commentary, Lorscheider acknowledges the importance of comorbidity management in PwMS but challenges its classification as disease-modifying. It argues that DMTs, by definition, must alter disease progression through direct effects on pathophysiology, a criterion that comorbidity-targeted interventions do not currently meet. While hypertension, hyperlipidemia, and psychiatric disorders are linked to worse MS outcomes,^7^ existing evidence largely stems from observational studies, making causal inferences unreliable. The MS-STAT2 trial of simvastatin in secondary progressive MS, which failed to slow disability progression, exemplifies the complexity of these associations.^9^ Similarly, while some MS therapies improve coexisting autoimmune conditions, others, such as tumor necrosis factor (TNF) inhibitors, effective therapeutic option in rheumatoid arthritis, psoriasis, and inflammatory bowel disease can worsen MS, highlighting the need for caution.^10^ Given the limited evidence for direct neuroprotective effects, optimizing comorbidity treatment should be a component of holistic MS care but not be equated with disease modification. The primary focus should remain on halting disability progression through targeted MS-specific therapies.

In summary, there is broad consensus on the critical importance of comorbidity management in PwMS, which extends beyond the selection of appropriate DMTs and might necessitate the involvement of multiple specialists. While the MS specialist remains central to patient care, an integrated care model, incorporating a multidisciplinary team to support MS management, should be prioritized. However, implementing such a paradigm shift presents significant challenges in terms of economic, human, and financial resources. To optimize treatment strategies and establish clear priorities for effective intervention, high-quality data are needed to guide evidence-based decision-making.
